# Obesity, Ethnicity, and Risk of Critical Care, Mechanical Ventilation, and Mortality in Patients Admitted to Hospital with COVID‐19: Analysis of the ISARIC CCP‐UK Cohort

**DOI:** 10.1002/oby.23178

**Published:** 2021-05-14

**Authors:** Thomas Yates, Francesco Zaccardi, Nazrul Islam, Cameron Razieh, Clare L. Gillies, Claire A. Lawson, Yogini Chudasama, Alex Rowlands, Melanie J. Davies, Annemarie B. Docherty, Peter J. M. Openshaw, J. Kenneth Baillie, Malcolm G. Semple, ISARIC4C investigators, Kamlesh Khunti

**Affiliations:** ^1^ Diabetes Research Centre University of Leicester Leicester General Hospital Leicester UK; ^2^ National Institute for Health Research (NIHR) Leicester Biomedical Research Centre (BRC) University Hospitals of Leicester NHS Trust and University of Leicester Leicester UK; ^3^ Leicester Real World Evidence Unit Diabetes Research Centre University of Leicester Leicester UK; ^4^ Clinical Trial Service Unit and Epidemiological Studies Unit (CTSU) Nuffield Department of Population Health University of Oxford Oxford UK; ^5^ Medical Research Council Epidemiology Unit University of Cambridge Cambridge UK; ^6^ Centre for Medical Informatics Usher Institute University of Edinburgh Edinburgh UK; ^7^ Intensive Care Unit Royal Infirmary Edinburgh Edinburgh UK; ^8^ National Heart and Lung Institute Imperial College London London UK; ^9^ Roslin Institute University of Edinburgh Edinburgh UK; ^10^ NIHR Health Protection Research Unit in Emerging and Zoonotic Infections and Institute of Translational Medicine, Faculty of Health and Life Sciences University of Liverpool Liverpool UK; ^11^ Respiratory Medicine Alder Hey Children’s Hospital Institute in The Park University of Liverpool Alder Hey Children’s Hospital Liverpool UK; ^12^ NIHR Applied Research Collaboration – East Midlands (ARC‐EM) University Hospitals of Leicester NHS Trust and University of Leicester Leicester UK

## Abstract

**Objective:**

The aim of this study was to investigate the association of obesity with in‐hospital coronavirus disease 2019 (COVID‐19) outcomes in different ethnic groups.

**Methods:**

Patients admitted to hospital with COVID‐19 in the United Kingdom through the Clinical Characterisation Protocol UK (CCP‐UK) developed by the International Severe Acute Respiratory and emerging Infections Consortium (ISARIC) were included from February 6 to October 12, 2020. Ethnicity was classified as White, South Asian, Black, and other minority ethnic groups. Outcomes were admission to critical care, mechanical ventilation, and in‐hospital mortality, adjusted for age, sex, and chronic diseases.

**Results:**

Of the participants included, 54,254 (age = 76 years; 45.0% women) were White, 3,728 (57 years; 41.1% women) were South Asian, 2,523 (58 years; 44.9% women) were Black, and 5,427 (61 years; 40.8% women) were other ethnicities. Obesity was associated with all outcomes in all ethnic groups, with associations strongest for black ethnicities. When stratified by ethnicity and obesity status, the odds ratios for admission to critical care, mechanical ventilation, and mortality in black ethnicities with obesity were 3.91 (3.13‐4.88), 5.03 (3.94‐6.63), and 1.93 (1.49‐2.51), respectively, compared with White ethnicities without obesity.

**Conclusions:**

Obesity was associated with an elevated risk of in‐hospital COVID‐19 outcomes in all ethnic groups, with associations strongest in Black ethnicities.

**Study Importance**

**What is already known?**

► People of South Asian or Black ethnic origin have been shown to have a higher risk of infection, severe disease, and coronavirus disease 2019 (COVID‐19) mortality compared with those of White ethnicities.
► Obesity is an established risk factor for COVID‐19 outcomes, but less is known about whether ethnicity acts to modify the strength of association observed with obesity or whether the risk remains consistent across ethnic groups.

**What does this study add?**

► Compared with White individuals without obesity, all other combinations of obesity and ethnicity had a higher risk of admission to critical care, receiving mechanical ventilation, or mortality in those admitted to hospital with COVID‐19. However, the risk of all outcomes was greatest in those of Black ethnicity with obesity.

**How might these results change the direction of research or the focus of clinical practice?**

► Black ethnic groups with obesity represent a particularly high‐risk group of patients, with implications for targeted public health and vaccination strategies and for identifying those most likely to suffer severe outcomes once admitted to hospital.

## Introduction

Obesity and ethnicity are well‐described risk factors for coronavirus disease 2019 (COVID‐19) outcomes (1, 2, 3, 4, 5, 6, 7). People of South Asian or Black ethnic origin, in particular, have been shown to carry a higher risk of infection, severe disease, and COVID‐19 mortality compared with those of White ethnicities (1, 2, 3, 4, 5). In addition, individuals with obesity have around twice the risk of severe outcomes or mortality compared with normal‐weight individuals (6, 7). However, although obesity and ethnicity were shown to be independent of each other as risk factors for COVID‐19 outcomes, less is known about whether ethnicity acts to modify the strength of association observed with obesity or whether the risk remains consistent across different ethnic groups.

The hypothesis that ethnicity may modify associations between obesity and COVID‐19 outcomes is drawn from previous research, suggesting that the dose–repose relationship between levels of obesity and cardiometabolic health is steeper in minority ethnic communities compared with White populations: indeed, the higher the BMI, the greater the difference in health outcomes between minority ethnic groups and White Europeans (8, 9, 10, 11, 12). As cardiometabolic diseases are known risk factors for COVID‐19 outcomes (13, 14, 15, 16), it is possible that obesity may also act as a particularly important risk factor for severe COVID‐19 outcomes in minority ethnic communities. Early research supports this hypothesis, in which the risk of severe acute respiratory syndrome coronavirus 2 infection, severe disease, and COVID‐19 mortality in minority ethnic communities has been shown to be magnified in the presence of obesity (17, 18), although this has not been confirmed in all studies (19). However, evidence to date is preliminary and based on small cohorts with a limited number of outcomes, with minority ethnic groups analyzed as one category. As minority ethnic groups cover heterogeneous populations, it remains uncertain whether associations between obesity and COVID‐19 outcomes differ in all minority ethnic groups or how they apply to national in‐hospital settings.

Investigating whether obesity is a stronger risk factor for in‐patient outcomes in specific minority ethnic groups will help inform public health and vaccination strategies aimed at identifying and targeting patients at greatest risk along with informing in‐hospital clinical decision‐making. In this view, we investigated associations of obesity and ethnicity with in‐hospital critical care and mortality outcomes in patients admitted with COVID‐19 using data from the Clinical Characterisation Protocol UK (CCP‐UK), a preparedness protocol for severe emerging diseases developed by the International Severe Acute Respiratory and emerging Infections Consortium (ISARIC) cohort (20). Previous ISARIC CCP‐UK publications have helped characterize in‐hospital patients admitted with COVID‐19 (21) and have shown a greater risk of in‐hospital outcomes with both obesity and ethnicity (21) (Harrison et al. SSRN, doi:10.2139/ssrn.3618215, unpublished data) during early phases of the pandemic. Having previously established independent associations, this paper investigates their interaction.

We hypothesized that obesity will be a stronger risk factor for in‐hospital outcomes within some minority ethnic groups.

## Methods

### Cohort

The protocol, amendment history, case report form, information leaflets, consent forms, and details of the Independent Data and Material Access Committee for ISARIC CCP‐UK are available at https://isaric4c.net. The study was approved by the South Central ‐ Oxford C Research Ethics Committee in England (Reference 13/SC/0149) and by the Scotland Research Ethics Committee (Reference 20/SS/0028). For this study, we included participants with a coding of “Proven or high likelihood of infection with a pathogen of Public Health Interest,” reflecting that a preparedness protocol cannot assume a diagnostic test will be available for an emergent pathogen. From January 2020 onward, site training also emphasized the importance of only recruiting proven cases of COVID‐19. Participants were included in the analysis if information was available on hospital admittance date from the emergence of the COVID‐19 pandemic in the United Kingdom (UK) (February 6, 2020); a completed coding indicating that the hospital admission had been resolved through discharge or in‐hospital mortality; and ethnicity status. Data were available up until October 12, 2020.

Data were directly transcribed from routine health care into case report forms hosted on a Research Electronic Data Capture database (REDCap; https://projectredcap.org). Data collection was undertaken by research nurses, administrators, and medical students. Detailed demographic and clinical data were collected on admission, with follow‐up data on clinical care collected at day 3, 6, and 9. The outcome of hospital admission was coded on discharge or death.

#### Exposures

Obesity was coded as yes or no on assessment from the attending clinician. Clinical assessment was based on objective measurement of obesity, such as BMI (BMI ≥ 30 kg/m^2^) or abdominal girth, or on clinical judgment.

Ethnicity was transcribed from health care records. In order to be consistent with internationally applicable ethnicity definitions, ethnicity within ISARIC was classified as East Asian, South Asian, West Asian, Black, White, Latin American, aboriginal/First Nations, and other ethnic minority. For the purposes of this analysis and based on frequency, ethnicity was categorized as White, South Asian, Black, or other (East Asian, West Asian, Arab, Latin American, Aboriginal/First Nations, other).

#### Covariates

Age was measured to the nearest year based on the difference between date of birth and hospital admission date. Sex was coded as male or female. Chronic disease was based on clinician‐diagnosed status. In this study, we included diseases that have been consistently associated with COVID‐19 outcomes (13, 14, 15, 16): chronic cardiac disease (coronary artery disease, heart failure, congenital heart disease, cardiomyopathy, rheumatic heart disease), chronic kidney disease (diagnosed chronic kidney disease or estimated glomerular filtration rate <60 mL/min/1.73 m^2^), chronic pulmonary disease (chronic obstructive pulmonary disease [chronic bronchitis, emphysema], cystic fibrosis, bronchiectasis, interstitial lung disease, preexisting requirement for long‐term oxygen therapy), diabetes (type 1 or 2), and malignant neoplasm (current solid organ or hematological malignancy). In‐hospital treatment for COVID‐19 was coded at discharge or death. The two main treatment types of oral or intravenous corticosteroids (including dexamethasone) and antivirals were included in this analysis.

#### Outcome

The main outcomes were admission to a critical care facility (intensive care unit [level 3] or high‐dependency unit [level 2]), any deployed usage of a mechanical ventilation procedure (tracheal intubation or tracheostomy), and in‐hospital mortality.

### Statistical analysis

We use logistic regression to quantify associations between obesity and outcomes stratified by ethnicity: odds ratios (ORs) were adjusted for age, sex, and the presence of comorbidities (diabetes, chronic heart disease, chronic kidney disease, chronic pulmonary disease, and cancer). Stratified analysis was undertaken to assess whether the pattern of association of obesity with assessed outcomes in each ethnic group was consistent across strata of age (<70 years, ≥70 years), sex and presence of any chronic disease (defined as diabetes, chronic heart disease, chronic kidney disease, chronic pulmonary disease, and cancer).

To further assess the pattern of associations for both obesity and ethnicity, we defined mutually exclusive groups of ethnicity and obesity and estimated ORs in each category compared with White individuals without obesity (reference group). Sensitivity analysis was undertaken to assess whether the association between mutually exclusive groups of ethnicity and obesity with mortality were independent of in‐hospital corticosteroid and antiviral treatments.

In order to account for missing obesity and covariate data, all analysis was conducted using multiple imputation through the Markov chain Monte Carlo imputation algorithm across five iterations. Associations with obesity within each ethnicity were also derived using a complete case data set as a sensitivity analysis.

All analyses were conducted in SPSS version 26 (IBM Corp., Armonk, New York). Data are reported with 95% CI unless reported otherwise; *P* < 0.05 was considered significant (e.g., in which the 95% CI does not cross the null).

## Results

Of the 65,932 individuals included in this analysis, 54,254 (82.3%) were White, 3,728 (5.7%) South Asian, 2,523 (3.8%) Black, and 5,427 (8.8%) from other ethnic minorities: Table 1 shows the characteristics of the cohort. Compared with White individuals, all minority ethnic groups were younger. Black ethnicities had the highest frequency of coded obesity (13.0%), although there was no difference compared with White individuals when accounting for differences in age and sex (OR 1.00; 95% CI: 0.88‐1.13). Conversely, age and sex adjusted frequencies were lower in South Asian (0.84; 95% CI: 0.76‐0.94) and other minority ethnic groups (0.76; 95% CI: 0.69‐0.84) than in White individuals. When adjusted for age and sex, all minority ethnic groups had a higher risk of in‐hospital outcomes, with associations persisting after further adjustment for obesity and chronic disease (Table 2). For example, compared with White ethnicities, South Asian (1.27; 95% CI: 1.17‐1.38), Black (1.19; 95% CI: 1.08‐1.32), and other ethnicities (1.12; 95% CI: 1.04‐1.21) had a higher risk of mortality.

**TABLE 1:** Cohort characteristics

	White (*n* = 54,254)		South Asian (*n* = 3,728)		Black (*n* = 2,523)		Other (*n* = 5,427)	
*Categorical variables*	**Number**	**%**	**Number**	**%**	**Number**	**%**	**Number**	**%**
**Sex**								
**Men**	29,817	55.0	2,186	58.6	1,393	55.2	3,205	59.1
**Women**	24,340	44.9	1,536	41.2	1,128	44.7	2,213	40.8
**Missing**	97	0.2	6	0.2	2	0.1	9	0.2
**Chronic heart disease**								
**No**	33,016	60.9	2,630	70.5	1963	77.8	3,915	72.1
**Yes**	18,428	34.0	772	20.7	380	15.1	1,080	19.9
**Missing**	2,810	5.2	326	8.7	180	7.1	432	8.0
**Chronic pulmonary disease**								
**No**	40,846	75.3	3,123	83.8	2,187	86.7	4,508	83.1
**Yes**	10,403	19.2	240	6.4	145	5.7	482	8.9
**Missing**	3,005	5.5	365	9.8	191	7.6	437	8.1
**Diabetes**								
**No**	41,796	77.0	2,362	63.4	1,744	69.1	3,858	71.1
**Yes**	7,604	14.0	852	22.9	505	20.0	953	17.6
**Missing**	4,854	8.9	514	13.8	274	10.9	616	11.4
**Chronic kidney disease**								
**No**	41,688	76.8	2,874	77.1	1944	77.1	4,339	80.0
**Yes**	9,380	17.3	495	13.3	393	15.6	633	11.7
**Missing**	3,186	5.9	359	9.6	186	7.4	455	8.4
**Cancer**								
**No**	44,879	82.7	3,222	86.4	2,153	85.3	4,621	85.1
**Yes**	5,823	10.7	126	3.4	175	6.9	327	6.0
**Missing**	3,552	6.5	380	10.2	195	7.7	479	8.8
**Antiviral treatment**								
**No**	48,286	89.0	3,096	83.0	2,086	82.7	4,558	84.0
**Yes**	2,914	5.4	309	8.3	251	9.9	481	8.9
**Missing**	3,054	5.6	323	8.7	186	7.4	388	7.1
**Corticosteroid treatment**								
**No**	43,005	79.3	2,682	71.9	1995	79.1	4,135	76.2
**Yes**	8,108	14.9	715	19.2	340	13.5	883	16.3
**Missing**	3,141	5.8	331	8.9	2,335	92.5	409	7.5
**Obesity**								
**No**	40,399	74.5	2,597	69.7	1,815	71.9	4,019	74.1
**Yes**	5,363	9.9	404	10.8	328	13.0	543	10.0
**Missing**	8,492	15.7	727	19.5	380	15.1	865	15.9
**Mortality**								
**No**	37,236	68.6	2,845	76.3	1932	76.6	4,153	76.5
**Yes**	17,018	31.4	883	23.7	591	23.4	1,274	23.5
**Missing**	0	0	0	0	0	0	0	0
**Mechanical ventilation**								
**No**	50,349	92.8	3,087	82.8	2,056	81.5	4,499	82.9
**Yes**	3,278	6.0	474	12.7	404	16.0	816	15.0
**Missing**	627	1.2	167	4.5	63	2.5	112	2.1
**Critical care**								
**No**	47,590	87.7	2,796	75.0	1,895	75.1	4,151	76.5
**Yes**	6,132	11.3	773	20.7	570	22.6	1,173	21.6
**Missing**	532	1.0	159	4.3	58	2.3	103	1.9
*Continuous variables*	**Median**	**Interquartile range**	**Median**	**Interquartile range**	**Median**	**Interquartile range**	**Median**	**Interquartile range**
**Age (y)**	76	63‐85	59	44‐73	59	47‐75	61	47‐76

**TABLE 2:** The risk of critical care admission, mechanical ventilation, and in‐hospital mortality in minority ethnic patients compared with White patients

In‐hospital outcome	White	South Asian	Black	Other
***Model 1***				
**Critical care admission**	Reference	1.40 (1.28‐1.53)	1.66 (1.49‐1.83)	1.53 (1.42‐1.65)
**Mechanical ventilation**	Reference	1.57 (1.40‐1.76)	2.17 (1.94‐2.44)	1.99 (1.82‐2.17)
**Mortality**	Reference	1.25 (1.15‐1.46)	1.18 (1.06‐1.30)	1.09 (1.02‐1.17)
***Model 2***				
**Critical care admission**	Reference	1.37 (1.26‐1.50)	1.58 (1.43‐1.75)	1.51 (1.40‐1.62)
**Mechanical ventilation**	Reference	1.49 (1.33‐1.68)	2.03 (1.80‐2.28)	1.91 (1.75‐2.09)
**Mortality**	Reference	1.27 (1.17‐1.38)	1.19 (1.08‐1.32)	1.12 (1.04‐1.21)

Data are given as odds ratio (95% CI). Model 1 is adjusted for age and sex. Model 2 is adjusted for age, sex, obesity, diabetes, chronic heart disease, chronic kidney disease, chronic pulmonary disease, and cancer.

**Figure 1:**
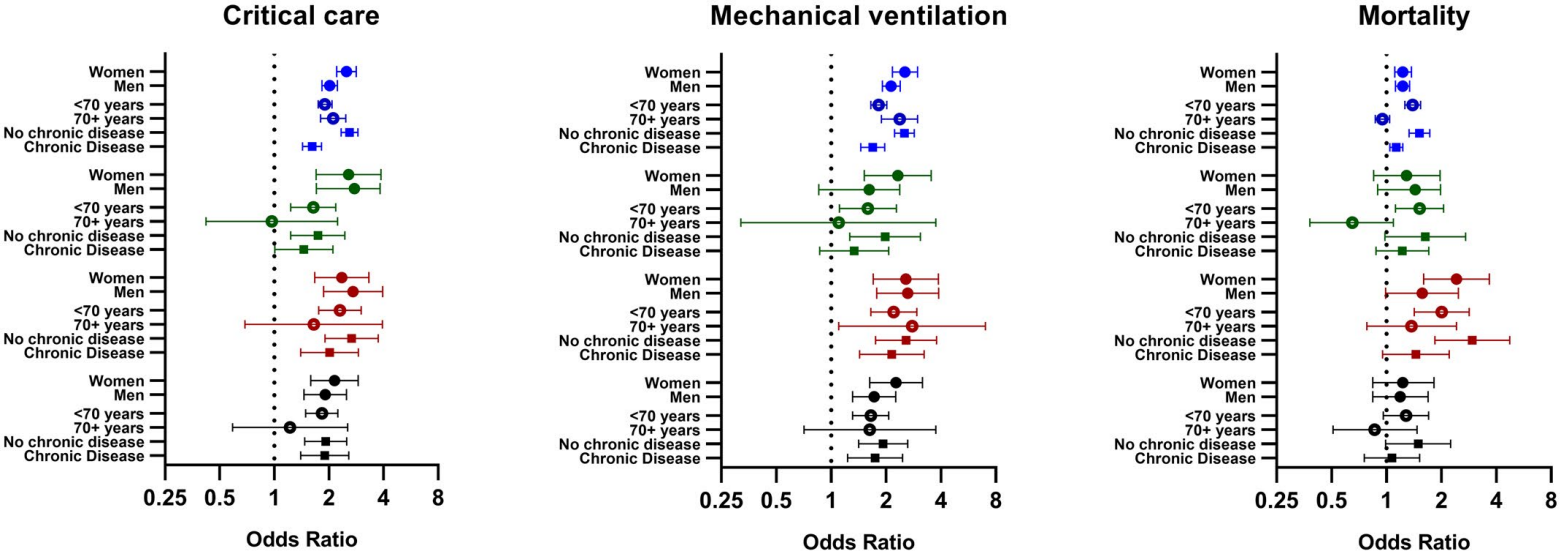
Associations of obesity (compared with those without obesity) with critical care, mechanical ventilation, and mortality for each ethnicity stratified by age, sex, and chronic disease. Error bars display 95% CI. Sex strata adjusted for age, diabetes, chronic heart disease, chronic kidney disease, chronic pulmonary disease, and cancer. Age strata adjusted for sex, diabetes, chronic heart disease, chronic kidney disease, chronic pulmonary disease, and cancer. Chronic disease strata adjusted for age and sex.

Obesity was consistently associated with admission to critical care, mechanical ventilation, and mortality in all ethnic groups (Table 3). However, for all outcomes, the association with obesity was strongest in Black ethnicities. For example, in White ethnicities, the OR for mortality in those with obesity compared with those without obesity was 1.23 (95% CI, 1.15‐1.32) whereas it was 1.98 (95% CI: 1.46‐2.68) for Black ethnicities. The association between obesity and in‐hospital mortality in South Asian (1.34; 95% CI: 1.03‐1.76) and other (1.22; 95% CI: 0.91‐1.62) minority ethnic groups was similar in magnitude to White ethnicities (Table 3). The pattern of association of obesity within each ethnic group was the same when the analysis was restricted to a complete case data set (Supporting Information Table S1). Associations within each ethnic group were also similar in men and women (Figure 1) but tended to be weaker in those with chronic disease or older (≥70 years) adults, particularly for mortality (Figure 1), in which no associations were seen in older adults for any ethnicity. The strongest association for obesity with mortality within assessed strata was observed in Black ethnicities without coexisting chronic disease (OR = 2.95; 95% CI: 1.84‐4.74) (Figure 1).

**TABLE 3:** Risk of COVID‐19 outcomes in those with obesity compared with the reference of those without obesity when stratified by ethnicity

In‐hospital outcome	White	South Asian	Black	Other
***Model 1***				
**Critical care admission**	2.07 (1.91‐2.24)	1.63 (1.26‐2.11)	2.42 (1.90‐3.09)	1.94 (1.61‐2.34)
**Mechanical ventilation**	2.11 (1.93‐2.32)	1.66 (1.18‐2.34)	2.44 (2.15‐3.31)	1.91 (1.55‐2.34)
**Mortality**	1.30 (1.22‐1.38)	1.42 (1.09‐1.85)	1.98 (1.46‐2.68)	1.29 (0.97‐1.70)
***Model 2***				
**Critical care admission**	2.20 (2.03‐2.38)	1.72 (1.32‐2.26)	2.50 (1.95‐3.20)	2.00 (1.66‐2.42)
**Mechanical ventilation**	2.27 (2.06‐2.49)	1.79 (1.27‐2.52)	2.56 (1.95‐3.37)	1.92 (1.56‐2.37)
**Mortality**	1.23 (1.15‐1.32)	1.34 (1.03‐1.76)	1.98 (1.46‐2.68)	1.22 (0.91‐1.62)

Data are given as odds ratio (95% CI). Reference group is those without obesity within each ethnic strata. Model 1 is adjusted for age and sex. Model 2 is adjusted for age, sex, diabetes, chronic heart disease, chronic kidney disease, chronic pulmonary disease, and cancer.

**Figure 2:**
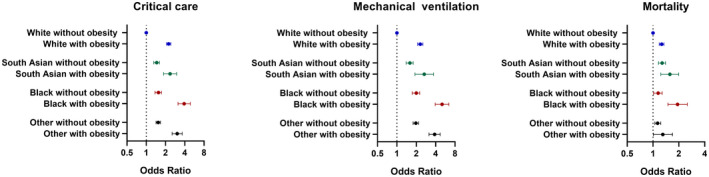
Risk of admittance to critical care, mechanical ventilation, and mortality across categories of obesity and ethnicity compared with White individuals without obesity. Error bars display 95% CI. Adjusted for age, sex, diabetes, chronic heart disease, chronic kidney disease, chronic pulmonary disease, and cancer.

Figure 2 shows the association of mutually exclusive categories of obesity and ethnicity with outcomes. Compared with White ethnicities without obesity, all other combinations of obesity and ethnicity had a higher risk of admission to critical care or having mechanical ventilation, with obesity having a stronger association with risk than ethnicity. For example, the OR of admission to a critical care facility in Black ethnicities without obesity was 1.54 (95% CI: 1.38‐1.73) compared with White ethnicities without obesity, whereas the OR in Black ethnicities with obesity was 3.91 (95% CI: 3.13‐4.88). The same comparisons for mechanical ventilation were 1.99 (95% CI: 1.74‐2.27) and 5.03 (95% CI: 3.94‐6.63), respectively (Figure 2). South Asian and other minority ethnic groups without obesity had a similar risk to Black ethnicities without obesity, but the risk in those with obesity was less pronounced. For mortality in those without obesity, risk was marginally higher for Black (1.14; 95% CI: 1.02‐1.28), South Asian (1.27; 95% CI: 1.16‐1.40) and other (1.13; 95% CI: 1.05‐1.23) minority ethnic groups (Figure 2). When considering patients with obesity, the risk of mortality was elevated in all ethnic groups; those with Black ethnicity and obesity had the greatest risk (1.93; 95% CI: 1.49‐2.51) (Figure 2). The pattern and strength of association of ethnicity and obesity with mortality was not affected by further adjusting for in‐hospital corticosteroid and antiviral treatments (Supporting Information Figure S1).

## Discussion

In this large national study of patients hospitalized with COVID‐19, obesity was associated with an increased risk of admission to critical care, receiving mechanical ventilation, and in‐hospital mortality in all ethnic groups, with associations strongest in those under 70 years of age or without other chronic diseases. However, the risk was consistently strongest in Black ethnicities with obesity when compared with all other ethnic and obesity groupings, with a four to five times greater risk of admission to critical care or receiving mechanical ventilation and around twice the risk of in‐hospital mortality compared with White ethnicities without obesity. The association of obesity with in‐hospital outcomes was stronger than that of ethnicity; in individuals without obesity, ethnicity was only marginally associated with in‐hospital mortality.

This study extends early preliminary findings from two different early analyses from the UK Biobank community cohort involving 1,087 positive cases and 189 deaths, in which obesity was a stronger predictor of positivity and COVID‐19 mortality in non‐White ethnicities (17, 18). The present study suggests that in patients admitted to hospital, a clinical coding of obesity is a stronger risk factor in Black ethnic groups specifically rather than for minority ethnic groups in general. Obesity in South Asian and Other ethnic minority groups carried a similar level of risk as it did for White ethnicities. There are several hypothesized mechanisms that may explain these findings. Obesity has been hypothesized to increase the risk of severe COVID‐19 through mechanisms linked to restricted pulmonary function and chronic inflammation (2, 22, 23, 24); adipose tissue has also been suggested as a viral reservoir (23). Patients from Black ethnicities may have greater inflammatory responses to infection (24, 25, 26), and excessive systemic innate inflammation due to obesity (“adipositis”) is associated with susceptibility to other infectious diseases (27). Because both clinical and genetic evidence strongly suggests that inflammatory processes drive mortality in COVID‐19 (28, 29), these mechanisms may place Black ethnicities with obesity at a higher risk of organ failure and death. It is also possible that sociodemographic factors, such as greater levels of deprivation and discrimination, may also act through a host of different mechanisms to lower resilience to infection or delay admission to hospital (24), which again could become more severe in the presence of obesity. However, further research is required to disentangle the mechanisms behind the observations reported in this paper.

Associations with obesity in each ethnic group were consistent across men and women but tended to be weaker in older (≥70 years) adults or those with coexisting chronic disease, particularly for the outcome of mortality, in which no association between obesity and mortality was seen in any ethnic group in older adults. Although there was a high level of uncertainty in older minority ethnic populations due to a younger average age and the resulting limited sample of older adults, the findings are consistent with another in‐hospital study in which associations of BMI with mortality or mechanical ventilation were largely attenuated in those over 70 years of age (30). Therefore, the findings from this study may only be generalizable to those under 70 years of age, with ethnic specific associations in older adults needing further investigation. Obesity may also be a particularly strong risk factor for severe COVID‐19 outcomes in otherwise healthy adults, where the highest odds of COVID‐19 mortality with obesity was seen in Black ethnicities without other coexisting chronic diseases.

Strengths of this analysis include the large multisite national sample with data captured from clinical records by trained individuals using standardized operating procedures, allowing the largest analysis of COVID‐19 outcomes with obesity and ethnicity to date. However, there are some important limitations. Obesity was defined through clinician assessment, with the prevalence an underestimate compared with levels that would have been expected based on population estimates (31). Thus, the coding of obesity possibly reflects more extreme phenotypes of obesity that are likely to prompt a clinical coding, and it is unknown whether this procedure was biased by ethnicity. However, the pattern of obesity prevalence in this sample is broadly consistent with national survey data for overweight and obesity prevalence (32), with rates higher in Black ethnic groups but lower in other minority ethnic groups compared with White ethnicities. It is also important to note that, in order to inform clinical care, analyzed risk factors need to reflect data that are readily available to treating clinical staff through clinical records. Therefore, the coding of obesity in this study may have real‐world utility as it corresponds to data collected within routine clinical care. Another potential limitation is that the coding of ethnicity was designed to be consistent with international definitions, rather than those designed for the UK populations. As such, it is acknowledged that the terms “Black,” “South Asian,” “White,” and “other” cover a wide range of different cultures and races. Consequently, our analyses may have masked important differences between further stratified ethnic groups. However, comparisons using these broad ethnic groupings are informative for understanding initial ethnic differences that can then be further and more granularly investigated. Finally, this analysis did not have access to potential sociodemographic confounders, such as deprivation, housing, or employment status. However, the purpose of this analysis was to highlight ethnic differences in the strength of obesity as a global risk factor for in‐hospital outcomes following admission with COVID‐19, rather than for supporting potential etiological conclusions around reducing levels of obesity per se.

In conclusion, although obesity was a consistent risk factor for adverse in‐hospital outcomes following admission with COVID‐19 within all ethnic groups, particularly for younger adults without coexisting chronic disease, the risk with obesity was greatest in Black ethnicities compared with other ethnic groups. Black ethnic groups with obesity therefore represent a particularly high‐risk group of patients with implications for targeted public health and vaccination strategies and for identifying those most likely to suffer severe outcomes once admitted to hospital.

## Funding agencies

This work was supported by the National Institute for Health Research (NIHR) Leicester Biomedical Research Centre (BRC), NIHR Applied Research Collaboration East Midlands (ARC EM), and a grant from the UK Research and Innovation ‐ Department of Health and Social Care (UKRI‐DHSC) COVID‐19 Rapid Response Rolling Call (MR/V020536/1 to TY); NIHR (award CO‐CIN‐01 to MGS); the Medical Research Council (MRC; grant MC_PC_19059 to MGS); and by the NIHR Health Protection Research Unit (HPRU) in Emerging and Zoonotic Infections at University of Liverpool. The funder/sponsor had no role in the design and conduct of the study; collection, management, analysis, and interpretation of the data; preparation, review, or approval of the manuscript; and decision to submit the manuscript for publication.

## Disclosure

KK is supported by the NIHR ARC EM and TY by the NIHR BRC. KK is Director for the University of Leicester Centre for Black and Minority Ethnic Health, trustee of the South Asian Health Foundation, national NIHR ARC lead for Ethnicity and Diversity, and a member of the Independent Scientific Advisory Group for Emergencies (SAGE) and Chair of the SAGE subgroup on ethnicity and COVID‐19. MGS is a member of SAGE COVID‐19. MGS reports grants from DHSC NIHR UK, grants from MRC UK, grants from HPRU in Emerging and Zoonotic Infections, University of Liverpool, during the conduct of the study, and other support from Integrum Scientific LLC, Greensboro, North Carolina, outside the submitted work. The other authors declared no conflict of interest.

## Supporting information

Supplementary Material
