# Supplementary material for: Obesity, Ethnicity, and Risk of Critical Care, Mechanical Ventilation, and Mortality in Patients Admitted to Hospital with COVID‐19: Analysis of the ISARIC CCP‐UK Cohort
Source: Obesity (Silver Spring). 2021 May 14;29(7):1223–30. doi: 10.1002/oby.23178 (PMC8251439; doi:10.1002/oby.23178)
Supplement: Supplementary file 1 — Supplementary Material [file OBY-29-1223-s001.docx]

|  |  | **White** | **South Asian** | **Black** | **Other** |
| --- | --- | --- | --- | --- | --- |
| **Complete Case (n = 51,593)** |  |  |  |  |  |
| Critical care admission |  | 2.46 (2.27, 2.656) | 1.85 (1.43, 2.39) | 2.75 (2.09, 3.62) | 2.16 (1.74, 2.67) |
| Invasive ventilation |  | 2.47 (2.24, 2.72) | 1.93 (1.43, 2.60) | 2.82 (2.08, 3.81) | 2.17 (1.71, 2.76) |
| Mortality |  | 1.32 (1.22, 1.42) | 1.41 (1.43, 1.91) | 2.37 (1.71, 3.28) | 1.26 (0.97, 1.64) |
|  |  |  |  |  |  |
| **Multiple imputation (65,932)** |  |  |  |  |  |
| Critical care admission |  | 2.20 (2.03, 2.38) | 1.72 (1.32, 2.26) | 2.50 (1.95, 3.20) | 2.00 (1.66, 2.42) |
| Invasive ventilation |  | 2.27 (2.06, 2.49) | 1.79 (1.27, 2.52) | 2.56 (1.95, 3.37) | 1.92 (1.56, 2.37) |
| Mortality |  | 1.23 (1.15, 1.32) | 1.34 (1.03, 1.76) | 1.98 (1.46, 2.68) | 1.22 (0.91, 1.62) |
|  |  |  |  |  |  |

**Supplementary Table S1: Association of obesity with in-hospital COVID-19 outcomes in the complete case vs the imputed dataset**

Data as odds ratio (95% CI). Reference group is those without obesity within each ethnic strata

Adjusted for age, sex, obesity, diabetes, chronic heart disease, chronic kidney disease, chronic pulmonary disease, cancer

**Supplementary Figure S1: Association of obesity and ethnicity with in-hospital COVID-19 mortality after further adjustment for in-hospital treatment**

**
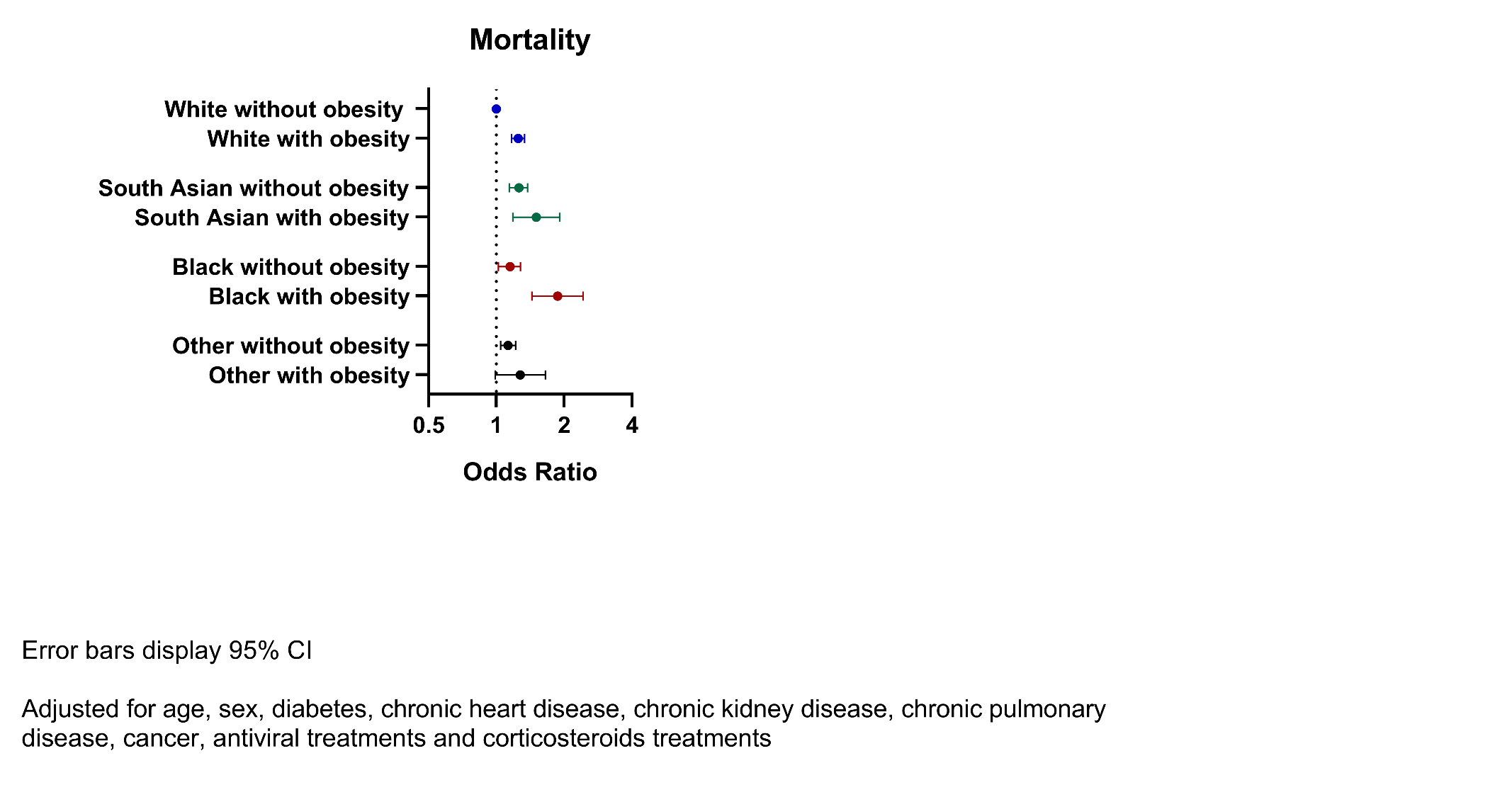
**
